# Diagnostic and prognostic value of procalcitonin among febrile critically ill patients with prolonged ICU stay

**DOI:** 10.1186/1471-2334-9-213

**Published:** 2009-12-22

**Authors:** Iraklis Tsangaris, Diamantis Plachouras, Dimitra Kavatha, George Michael Gourgoulis, Argirios Tsantes, Petros Kopterides, George Tsaknis, Ioanna Dimopoulou, Stylianos Orfanos, Evangelos Giamarellos-Bourboulis, Helen Giamarellou, Apostolos Armaganidis

**Affiliations:** 1 The 2nd Critical Care Department, Attikon University General Hospital, Medical School, University of Athens, 1 Rimini Str., 12462, Athens, Greece; 2 The 4th Department of Internal Medicine, Attikon University General Hospital, Medical School, University of Athens, 1 Rimini Str., 12462, Athens, Greece; 3 Laboratory of Haematology & Blood Bank Unit Attikon University General Hospital, Medical School, University of Athens, 1 Rimini Str., 12462, Athens, Greece

## Abstract

**Background:**

Procalcitonin (PCT) has been proposed as a diagnostic and prognostic sepsis marker, but has never been validated in febrile patients with prolonged ICU stay.

**Methods:**

Patients were included in the study provided they were hospitalised in the ICU for > 10 days, were free of infection and presented a new episode of SIRS, with fever >38°C being obligatory. Fifty patients fulfilled the above criteria. PCT was measured daily during the ICU stay. The primary outcome was proven infection.

**Results:**

Twenty-seven out of 50 patients were diagnosed with infection. Median PCT on the day of fever was 1.18 and 0.17 ng/ml for patients with and without proven infections (p < 0.001). The area under the curve for PCT was 0.85 (95% CI; 0.71-0.93), for CRP 0.65 (0.46-0.78) and for WBC 0.68 (0.49-0.81). A PCT level of 1 ng/mL yielded a negative predictive value of 72% for the presence of infection, while a PCT of 1.16 had a specificity of 100%. A two-fold increase of PCT between fever onset and the previous day was associated with proven infection (p 0.001) (OR = 8.55; 2.4-31.1), whereas a four-fold increase of PCT of any of the 6 preceding days was associated with a positive predictive value exceeding 69.65%. A PCT value less than 0.5 ng/ml on the third day after the advent of fever was associated with favorable survival (p 0.01).

**Conclusion:**

The reported data support that serial serum PCT may be a valuable diagnostic and prognostic marker in febrile chronic critically ill patients.

## Background

An increasing proportion of critically ill patients require prolonged intensive care unit (ICU) stay [1]. It is not unexpected that this group of patients is associated with increased risk for all kind of complications, adverse short-term outcomes, and increased consumption of ICU resources with infectious complications being always at the center of the discussion in this setting [2]. Although survivors of prolonged ICU stays have good long-term mortality outcome, ICU mortality remains high and severely affected by sepsis and its complications [3]. The presence of nosocomial infection and the number of infection episodes have been identified as the variables with the strongest association with prolonged hospital stay among ICU patients [4], and the effect of the most common ICU infections on duration of ICU stay has been well documented [5,6].

Patients with prolonged ICU stay are prone to be colonized and infected by multi-drug resistant bacteria. Furthermore these patients have often their metabolic and neurohormonal reserves exhausted, while their immune response might be severely affected [7]. The combination of the above makes the identification of a new infectious episode quite often a clinical challenge, while on the other hand these patients have many reasons to develop a systemic inflammatory response not associated with infection. Every intensivist is constantly dealing with these 'grey-zone' cases, fearing delayed diagnosis and treatment of a new infection on one hand, and excess antibiotic use, multi-resistance, and increased costs on the other.

Procalcitonin (PCT) has been proposed as a marker to differentiate sepsis from other non-infectious causes of SIRS. After the initial enthusiasm, many studies have provided conflicting results on the diagnostic accuracy of PCT in different patient settings including critically ill patients [8,9], and have highlighted the importance of integrating laboratory and clinical evaluation. On the other hand, PCT has been shown capable to guide antibiotic use in lower respiratory tract infection [10], community-acquired pneumonia [11] and, recently, in critically ill patients with sepsis [12].

Even though recently published studies [13,14] conducted in single centers with selected patient populations have shown the diagnostic value of PCT monitoring in the ICU setting, confirmatory research is urgently needed before the widespread adoption of this practice. In addition, it is unclear if the diagnostic accuracy of PCT diminishes in prolonged need for critical care probably due to intercurrent confounding conditions. Finally, the potential prognostic value of PCT measurements in this particular patient population has not been thoroughly studied.

The aim of the present study is to evaluate prospectively the diagnostic accuracy of PCT in ICU patients with prolonged stay (defined by an ICU length of stay ≥ 10 days), to compare it with other commonly used laboratory and clinical markers and to examine the value of serial PCT measurements in this setting.

## Methods

This observational cohort study was performed in accordance with the Declaration of Helsinki and was approved by the 'Attikon' University Hospital's review board. Informed consent was obtained from the patient's next of kin. All patients hospitalized in the ICU of Attikon University Hospital between November 1^st ^2007 and April 30^th ^2008 were assessed for eligibility and had their circulating PCT levels prospectively measured daily as long as they stayed in the ICU. Patients were included in the study provided they 1) were hospitalised in the ICU for more than 10 days, 2) were considered free of infection on admission to the study according to the evaluation of the treating physician and 3) presented a new episode of SIRS, with fever >38°C being an obligatory criterion.

Demographic data, comorbidities and clinical parameters including microbiology data were recorded upon study enrollment. Patients were considered as being colonized with multi-drug resistant (MDR) pathogens if at least one multi-drug resistant strain was isolated at the last anal swab or tracheal aspirate screening which is routinely performed twice weekly in our ICU. MDR bacteria were defined as methicillin-resistant *Staphylococcus aureus *or for Gram-negative bacilli, resistant against at least to two different classes of antibiotics such as penicillins (+beta-lactamase inhibitor), third-generation cephalosporins, fluoroquinolones or carbapenems. Severity indexes, including Acute Physiology and Chronic Health Evaluation (APACHE) II and Sequential Organ Failure Assessment (SOFA) scores were calculated at baseline, while SOFA was calculated on a daily basis. SIRS, sepsis, severe sepsis and septic shock were defined according to SCCM/ESICM/ACCP/ATS/SIS Consensus Conference [15]. The primary outcome was proven infection. The diagnosis of respiratory tract infections, urinary tract infections, bloodstream infections, and catheter-related infection followed the Centers for Disease Control definitions [16,17].

Blood samples were collected every morning between 8.00 and 9.00 a.m. Besides routine hematology and biochemistry panels, samples for C-reactive protein (CRP) and procalcitonin (PCT) were drawn daily. Samples for PCT were centrifuged at 3000 rpm for 10 minutes, and sera were stored at -80°C. For serum PCT measurement, a time-resolved amplified cryptate emission technology assay was used according to the manufacturer's instructions (Kryptor, Brahms, Hennigsdorf, Germany). The functional sensitivity of this assay is 0.06 ng/mL. Results for PCT were not made available to the attending clinicians.

### Statistics

Continuous variables were compared between the groups with and without proven bacterial infection by non-parametric Mann-Whitney U test. Receiver operator characteristic (ROC) curves were drawn for PCT, CRP and WBC count. Repeated measurements were compared between groups by General Linear Model. Differences with values of p less than 0.05 were considered statistically significant. Statistical analysis was performed using SPSS version 13.0 (SPSS Inc., Chicago, Ill).

## Results

During the study period 154 patients were screened for eligibility, and 50 patients fulfilled the inclusion criteria. The diagnostic work-up revealed 27 patients with proven infections. The general characteristics of study population with and without proven infection are shown in Table 1. Patients with and without proven infection differed significantly in age and severity of illness; the proportion of patients suffering a previous septic episode was not different between the two groups. WBC count, CRP and PCT in the groups with and without proven infection are presented in Table 2. Among the 27 patients with proven infection, the diagnosis was bloodstream infection in eight (30%), lung infection in 16 (59%) and abdominal infection in 3 (11%). Median (interquartile range) PCT concentrations were 15.7 ng/ml (26.6), 1.0 (2.4) and 1.0 respectively in these three groups. Excluding patients with blood stream infection, median PCT (IQR) values in patients without and with proven infection were 0.18 (0.39) and 1.00 (2.69) mg/L respectively (p 0.001).

**Table 1:** Demographic and clinical data of the patient population

	Proven infection (n = 27)	Unproven infection (n = 23)	P
Age, years	70 ± 12.1	56 ± 22.1	0.03

Gender (male)	20 (74%)	18 (78%)	NS

ICU stay duration until study enrollment	25 ± 12	29 ± 14	NS

APACHE II score on study enrollment	20.5 ± 4.4	15.3 ± 4.1	<0.001

SOFA score on day 1 (Day of fever)	10.1 ± 3	6.2 ± 2.4	0.004

Δ SOFA score	2.7 ± 1.9	0.6 ± 1	<0.001

28-day mortality	15/27 (56%)	7/23 (30%)	NS

Day 1 (Day of fever) status			

SIRS	1 (4%)	6 (26%)	NS

SIRS+New organ failure (besides circulation)	13 (48%)	14 (61%)	NS

Shock	13 (48%)	3 (13%)	0.07

Admission category			

Respiratory	7 (26%)	6 (26%)	NS

Cardiovascular	6 (22%)	3 (13%)	NS

Neurological	9 (33%)	10 (43%)	NS

Trauma/Surgical	5 (19%)	2 (9%)	NS

Co-morbidities			

Malignancy	2 (7%)	2 (9%)	NS

CRRT	8 (30%)	5 (22%)	NS

Steroids	4 (15%)	3 (13%)	NS

Previous septic episodes			NS

MDR pathogens in the last tracheal aspirate	27/27 (100%)	21/23 (91%)	NS

MDR pathogens in the last anal swab	27/27 (100%)	20/23 (87%)	NS

Proven infection	17/27(63%)	13/23(56%)	NS

Ventilator-associated pneumonia	16 (60%)	0	

Bloodstream infection	8 (30%)	0	

Abdominal infection	3 (10%)	0	

ICU: Intensive Care Unit, APACHE: Acute Physiology and Chronic Health Evaluation, SOFA: System Organ Failure Assessment, ΔSOFA: difference between Day 1 (fever) and Day 0 in SOFA score, SIRS: Systemic Inflammatory Response Syndrome, CRRT: Continuous Renal Replacement Therapy, MDR: Multi-Drug Resistant.

**Table 2:** Markers of infection in the groups with and without proven infection and diagnostic validity

	No proven infection	Proven infection	P	Sens	Spec	PPV	NPV	PLR	AUC
Patient *n *(%)	23 (46)	27 (54)							

WBC count (10^9^/μ) - Median (IQR)	8.4 (3.4)	14.2 (16.3)	0.03	0.66	0.45	0.76	0.62	2.72	0.68

CRP (mg/dl) - Mean (SD)	88.3 (64.0)	122.4 (66.6)	0.07	0.59	0.57	0.62	0.54	1.36	0.65

PCT (mg/L) - Median (IQR)	0.17 (0.39)	1.18 (5.8)	<0.001	0.70	0.73	0.75	0.68	2.62	0.85

CRP cut-off: 100; WBC cut-off: 12,000; PCT cut-off: 0.5

PPV: Positive Predictive Value, NPV: Negative Predictive Value, PLR: Positive Likelihood Ratio, AUC: Area Under Curve, WBC: White Blood Cells, IQR: Interquartile Range, CRP: C- reactive protein, SD: Standard Deviation, PCT: Procalcitonin.

**Figure 1:**
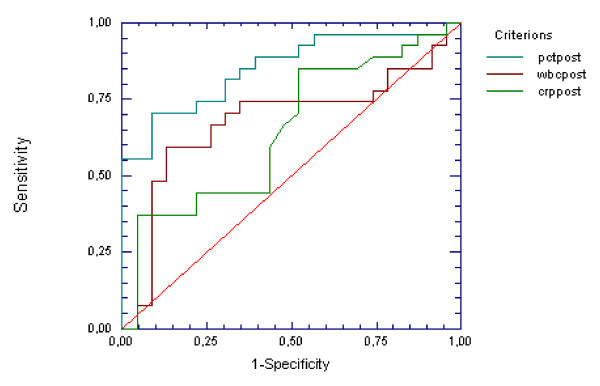
**ROC curves for PCT, CRP and WBC count for differentiation between proven and not-proven infections**. ROC: receiver operator characteristics, PCT: Procalcitonin, CRP: C- reactive protein, WBC: White Blood Cells.

The area under the ROC curve (95% CI) for PCT was 0.85 (0.71-0.93) (p < 0.0001), for CRP was 0.65 (0.46-0.78) (p NS) and for WBC count was 0.68 (0.49-0.81) (p 0.01) (Figure 1). A cut-off value for PCT of 0.15 ng/ml was characterized by sensitivity of 96%, while a cut-off of 1.16 ng/ml had a specificity of 100% for proven infection. For a cut-off value of 1 ng/ml, sensitivity was 70%, specificity 91%, and likelihood ratio for a positive test (LR+) was 8.1, positive predictive value (PPV) of 90% and negative predictive value (NPV) of 72%.

**Figure 2:**
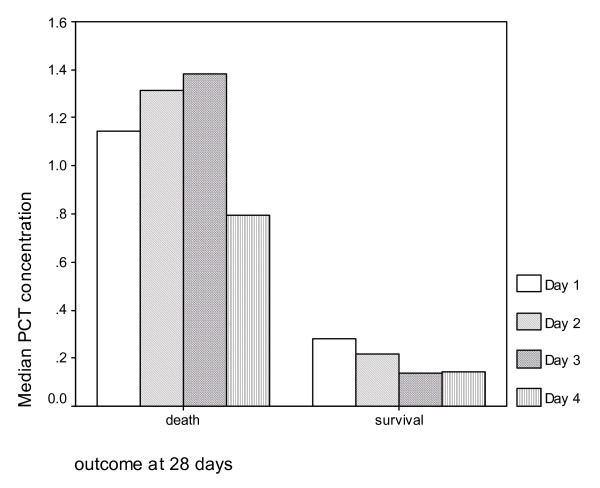
**Trend of PCT concentrations on sequential daily measurements between days 1 and 4 in patients with and without favorable outcome at 28 days**. PCT: Procalcitonin.

Median (IQR) PCT concentrations in the group of patients who survived at 28 days were 0.28 ng/ml (0.80) and in patients who did not survive were 1.07 (3.09) (p 0.004). Respective values for CRP were 89.5 (100.4) and 131.0 (106.5) in survivors and non-survivors respectively (p 0.01). Median PCT concentrations sequentially measured on days 1, 2, 3 and 4 tended to increase in non-survivors contrary to survivors (p 0.028) (Figure 2). Specifically, a concentration of PCT less than 0.5 ng/ml on the third day after the advent of fever was associated with favorable survival (p 0.01).

**Figure 3:**
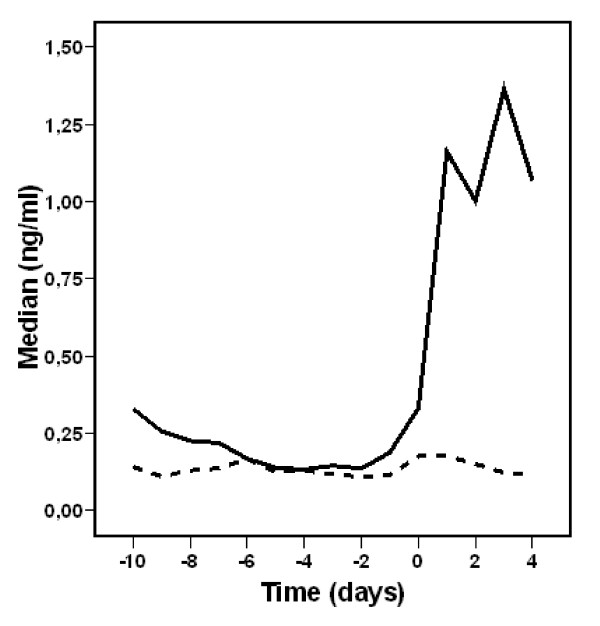
**PCT values for patients with (constant line) and without (dotted line) proven infection before and after the onset of fever (D1)**. PCT: Procalcitonin, D1: Day1 (day of fever onset).

PCT values before onset of fever for patients with and without proven infection are depicted at Figure 3. For the group with proven infection, the difference between the PCT value in the day of fever onset and any of the 10 preceding days was significant (P < 0.001 for any day compared to fever onset day). When patients without proven infection had their PCT value of the day of fever onset compared to the PCT of the 5 preceding days, the p value was 0.023, 0.045, 0.05, 0.013, and 0.030 for days 1, 2, 3, 4, and 5, respectively. A two-fold increase of PCT value between the day of fever onset and the previous day value was significantly associated with proven infection (p 0.001) (OR 8.55, 2.4-31.1) (Table 3). Sensitivity, specificity and positive predictive value of a four-fold increase between any PCT values of the 6 days preceding fever are shown at Table 4.

**Table 3:** Association between diagnostic markers and proven infection

Parameter	OR	95% CI	P
PCT > 0.5	8.6	2.4-31.0	0.001

CRP > 100	1.4	0.5-4.3	0.6

WBC > 12,000	9.7	2.3-40.8	0.001

SOFA > 7	5.4	1.6-18.0	0.006

PCT > 2 × D0	8.55	2.4-31.1	0.001

OR: Odds Ratio, CI: confidence interval, PCT: Procalcitonin, CRP: C - reactive protein, WBC: White Blood Cells, SOFA: Sequential Organ Failure Assessment.

**Table 4:** Validity of a four-fold increase of PCT value between day 1 (PCT1: day of fever onset) and the six previews days (PCT0 to PCT-5) for differentiating patients with and without proven infection

	Sensitivity	Specificity	PPV
PCT1-PCT0	59.26	86.95	84.20

			

PCT1-PCT(-1)	64	82.60	80

			

PCT1-PCT(-2)	68	77.27	77.27

			

PCT1-PCT (-3)	69.56	76.17	69.65

			

PCT1-PCT(-4)	66.67	80	77.77

			

PCT1-PCT(-5)	66.67	73.68	73.68

PPV: Positive Predictive Value, PCT: Procalcitonin

## Discussion

The main finding of the study was that PCT monitoring retains its diagnostic value even after a prolonged ICU stay, as it could differentiate a population of chronic critically ill patients with microbiologically proven sepsis from a similar population presenting merely with a new episode of SIRS and fever without proven infection. Regarding the primary outcome of proven infection, PCT performed better than other widely used infection markers, such as CRP and WBC count, in this population of patients. Another notable finding was that PCT monitoring possesses not only diagnostic but prognostic information as well; in fact, serial PCT measurements demonstrated an increasing tendency in non-survivors, while a PCT value of less than 0.5 ng/ml on the third day after the advent of fever was associated with a favorable survival outcome. Finally, it is interesting that every PCT value taken during the days preceding the advent of fever, and not only the previous day sample, carries potentially important predictive value. This could mean that serial PCT values do not need to be obtained on a daily basis, but twice or three times weekly, a finding with obvious economic implications.

Differentiating chronic critically ill patients with sepsis from patients with fever but without sepsis is notoriously difficult. Infections are not always associated with fever, the lack of positive culture results cannot exclude infection, and the traditional definition of SIRS has limited value because many patients might have SIRS criteria due to their status, whereas clinical evaluation remains sometimes the only rational approach. It is increasingly clear that none of the widely used biomarker for sepsis is perfect, but PCT performed much better than CRP and WBC count in chronic critically ill. This finding has been shown in other selected populations [18,19] but to our knowledge has never been demonstrated in chronic critically ill. In our cohort, PCT on the day of fever was significantly higher in patients with proven infection compared to the previous day value, while CRP was not. PCT was significantly higher even if we excluded blood stream infections, demonstrating a good performance in all other infections as well. A two-fold increase in previous day PCT was associated with infection and a four-fold increase of the PCT value of any of the 6 preceding days was associated with specificity exceeding 74% and a positive predictive value exceeding 70% for proven infection. On the other hand a PCT level of 1 ng/mL yielded a negative predictive value of 72% in our patients, while a PCT of 1.16 ng/ml had a specificity of 100%. Different PCT cut-off values in medical and surgical critically ill patients, not only at the ICU admission but also during the entire ICU stay have been demonstrated previously [20] and the need for cut-off points tailored to certain populations and conditions is increasingly recognized. Even previous episodes of sepsis, a common finding in chronic critically ill patients, seem to affect PCT performance [21]. Recent studies have upgraded the significance of PCT in guiding antibiotic use, based on its negative predictive value and the assumption that a patient with a low PCT does not have bacterial sepsis [10,11]. A recent trial conducted in septic patients, confirmed the potential of this approach in reducing antibiotic use. Unfortunately this elegant study focused on a population of patients that did not harbor the most 'difficult to treat' strains, bypassing the problem of multi-resistance.

Increasing serial PCT levels are associated with poor outcomes in patients with severe infections [22,23]. Jensen et al [24] demonstrated that when PCT increased the day following the first day after the blood level exceeded 1.0 ng/mL, the 90-day mortality was significantly higher. Charles et al found a significant decrease of PCT both between the second and third day, as well as on the fourth day after the onset of sepsis in a cohort of critically ill patients treated with appropriate antibiotic therapy [25]. The small sample size of the present study does not allow any association of PCT value with the appropriateness of therapy, but our finding are compatible with other studies [24,25] that demonstrate that a persistently high or increasing PCT value is an ominous prognostic sign.

What does our study offer to the field? Prolonged ICU stay is probably the most important determinant of ICU cost [26]. An important proportion of critically ill patients who survive their acute illness remain in a critical state requiring intensive care management for weeks to months. These patients are extremely prone to new infections both because they are constantly exposed to a high-risk microbiological environment and because their immune efficiency might be affected [27]. Furthermore our clinical and laboratory tools for diagnosing sepsis have been developed in different populations and have not been validated in chronic critically ill patients, a population of patients that is expected to increase in future. Although this study shows that PCT measurement can be used as a useful marker of infection in this setting, the introduction of PCT in the treatment algorithm of chronic critically ill patients requires further studies. Hopefully the ongoing intervention studies [28] will include a significant number of chronic critically ill patients, allowing reliable conclusions. Another potentially important finding of the study is that every PCT value taken during the days preceding the advent of fever, and not only the previous day sample, carries potentially important predictive value. This could mean that serial PCT values do not need to be obtained on a daily basis, but twice or three times weekly, a finding with obvious economic implications.

This study has certain limitations. It is a single-center study in a population of chronically ill patients and this could affect the generalization of the data, especially by the time that practice of critical care in these populations is not homogeneous and might have significant differences from site to site. Another limitation is that, almost all of our chronic patients have been colonized with gram-negative multi-resistant bacteria and the vast majority of proven infections (25/27) were of Gram-negative origin. Peak PCT response after Gram-negative challenge has been found higher in vitro [29] as well as in vivo [30]; therefore the performance of PCT in populations of critically ill patients exposed to other microbial populations should be investigated. Finally PCT values may not exclusively be related to the underlying infection but to coexisting organ failure as well; the small sample size of the study does not allow a thorough analysis of this issue.

## Conclusion

Serial serum PCT seems to be a marker capable of differentiating chronic critically ill patients with manifested infections compared to those presenting with fever of non-infectious origin, demonstrating both diagnostic and prognostic value. Although these observations support the existing literature conducted in other selected populations, further studies in big number are needed to describe the precise role of PCT in the management of these extremely complicated cases.

## Key messages

• PCT could differentiate patients with microbiologically proven sepsis among febrile critically ill patients with prolonged ICU stay.

• A two-fold increase of PCT value between the day of fever onset and the previous day is associated with proven infection in febrile critically ill patients with prolonged ICU stay.

• In chronic critically ill patients PCT performs better than other widely used infection markers, such as CRP and WBC count.

• PCT less than 0.5 ng/ml on the third day after the advent of fever was associated with favorable survival in febrile critically ill patients with prolonged ICU stay.

## Abbreviations

PCT: procalcitonin; ICU: Intensive Care Unit; SIRS: Systemic Inflammatory Response Syndrome; CRP: C - reactive protein; AUROCC: area under the receiver operating characteristic curve; CI: confidence interval; PPV: positive predictive value; NPV: negative predictive value; IQR: interquartile range; LPS: lipopolysaccharide; SOFA: Sequential Organ Failure Assessment.

## Competing interests

The authors declare that they have no competing interests.

## Authors' contributions

IT designed the study, analyzed the data and drafted the manuscript. DK, GMG, PK and GT collected the data and participated to their interpretation. DK, GMG, and AT managed the laboratory measurements. DP and EGB performed the statistical analysis. DP, AT, PK, ID, SO, EGB, HG and AA participated to critical revision of the manuscript. All authors read and approved the final manuscript.

## Pre-publication history

The pre-publication history for this paper can be accessed here:

http://www.biomedcentral.com/1471-2334/9/213/prepub
